# Acute Ethanol Exposure during Synaptogenesis Rapidly Alters Medium Spiny Neuron Morphology and Synaptic Protein Expression in the Dorsal Striatum

**DOI:** 10.3390/ijms23010290

**Published:** 2021-12-28

**Authors:** Erin Clabough, James Ingersoll, Tyler Reekes, Alyssa Gleichsner, Amy Ryan

**Affiliations:** 1 Department of Psychology, University of Virginia, Charlottesville, VA 22904, USA; 2 Department of Biology, Hampden-Sydney College, Hampden-Sydney, VA 23943, USA; ingersolljj@gmail.com (J.I.); treeke@lsuhsc.edu (T.R.); 3 Department of Pharmacology, Toxicology, and Neuroscience, Louisiana State University Health Sciences Center, Shreveport, LA 71104, USA; 4 Department of Biological Science, SUNY Plattsburgh, Plattsburgh, NY 12901, USA; aglei002@plattsburgh.edu (A.G.); amy.ryan@plattsburgh.edu (A.R.)

**Keywords:** environmental chemicals, maternal exposure, cognitive deficits, fetal alcohol spectrum disorder, dendritic morphology, striatum

## Abstract

Fetal alcohol spectrum disorders are caused by the disruption of normal brain development in utero. The severity and range of symptoms is dictated by both the dosage and timing of ethanol administration, and the resulting developmental processes that are impacted. In order to investigate the effects of an acute, high-dose intoxication event on the development of medium spiny neurons (MSNs) in the striatum, mice were injected with ethanol on P6, and neuronal morphology was assessed after 24 h, or at 1 month or 5 months of age. Data indicate an immediate increase in MSN dendritic length and branching, a rapid decrease in spine number, and increased levels of the synaptic protein PSD-95 as a consequence of this neonatal exposure to ethanol, but these differences do not persist into adulthood. These results demonstrate a rapid neuronal response to ethanol exposure and characterize the dynamic nature of neuronal architecture in the MSNs. Although differences in neuronal branching and spine density induced by ethanol resolve with time, early changes in the caudate/putamen region have a potential impact on the execution of complex motor skills, as well as aspects of long-term learning and addictive behavior.

## 1. Introduction

Ethanol is a known neurotoxin, yet drinking alcohol is an extremely common practice in our society. This consumption is particularly problematic when pregnant women drink alcohol, because ethanol can cause physical and neurological defects in a temporal and dose-dependent manner in a developing organism, possibly causing fetal alcohol spectrum disorders (FASD). Although many women are aware of the dangers of drinking while pregnant, they may be more likely to drink towards the end of their pregnancy, when the baby is further along in development and the perceived risk to the baby may be lower [1]. However, the critical period for the development of the central nervous system spans the course of human gestation, so there is no safe amount to drink at any time during pregnancy.

Animal models can replicate many of the physical, neurological, and behavioral signs of FASD using either a chronic or acute ethanol treatment paradigm. Chronic ethanol exposure can be either a low or high dose and chronic mouse models of FASD have shown that prenatal ethanol exposure can alter motor functioning, learning and memory, and social behavior. Neuroanatomically, the effects of moderate chronic ethanol exposure throughout gestation in rats can be seen in the striatum, drastically reducing the branching of dendrites and the lengths of medium spiny neurons (MSN) in the nucleus accumbens in adult offspring [2].

In contrast, the acute treatment model is nearly always high dose, and mimics the conditions of a binge drinking session. Fewer research studies have used an acute ethanol exposure model to study behavior, so the effects of acute ethanol exposure remain somewhat less certain and are highly dependent on the timing of the dose. However, acute ethanol exposure can allow the researcher to identify subtler behavioral changes that may occur in the absence of chronic exposure, as well as target more specific developmental periods, such as neurulation, neuronal migration, or synaptogenesis.

The neurodevelopment that occurs between human gestational months 7 and 9 is roughly equivalent to the processes that occur in mice and rats from birth until 2 weeks postnatally. This time period is most sensitive to neurodegeneration due to ethanol exposure, likely due to simultaneous ongoing synaptogenesis [3,4]. In mice, an acute ethanol exposure during synaptogenesis creates apoptotic neurodegeneration within 24 h in brain regions that include specific areas of the cortex (frontal, cingulate, parietal, temporal, and retrosplenial cortex), as well as the hippocampal formation (CA1 and subiculum), anterior thalamus, and mammillary bodies, and striatum [3,4,5]. In the striatum, specifically, acute administration of ethanol between postnatal days 5–7 causes a significant decrease in caudate/putamen MSN dendritic branch points, spine density, and a reduction in the size of the soma in comparison with wild-type controls at P30 [6]; however, the immediate impact on MSN morphology has not been examined.

Previous work from our lab found that acute ethanol treatment during the early postnatal period in mice causes an adult memory retention deficit in a spatial memory task [7]. The striatum—particularly the caudate/putamen—is very important for properly learning and executing complex spatial tasks, and improper striatal functioning may contribute to the learning and memory deficits we observed in the Barnes maze in adulthood. The majority of neurons (90–95%) in the caudate/putamen are inhibitory medium spiny neurons (MSNs). These neurons have extensive dendritic trees that receive cortical input, as well as input from other brain regions. Ethanol exposure can change MSN GABAergic synaptic transmission (reviewed in [8]). Alterations in neurotransmitter levels and their receptors can occur in sync with morphological changes in synapses and dendrites. This might be a possible explanation for some cognitive function deficits caused by ethanol exposure [9].

To investigate ethanol-induced alterations in MSNs, we administered acute high doses of ethanol during synaptogenesis in mice and assessed the subsequent neuronal morphology and dendritic spine changes during several different developmental timepoints (early postnatal, adolescence, and adulthood). Molecular analysis of proteins associated with the stress response (Heat Shock Protein-70; HSP70) and neuronal pruning by microglia (fractalkine) were also analyzed immediately after ethanol administration, along with levels of the glutamatergic synapse marker Post-Synaptic Density-95 (PSD-95).

## 2. Results

### 2.1. Neuronal Morphology following Ethanol Administration

**Figure 1:**
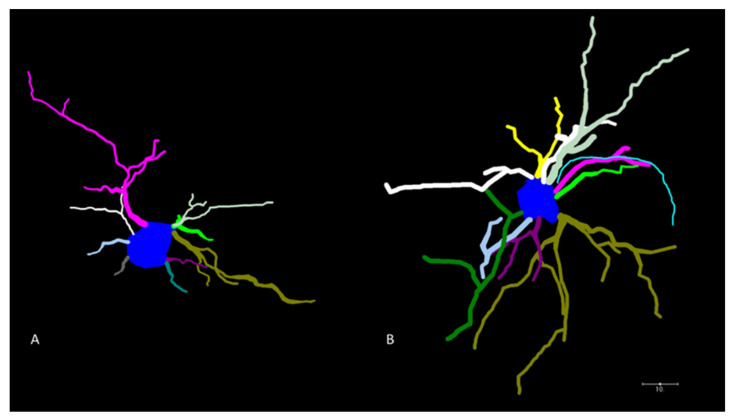
Golgi-stained MSNs in the caudate/putamen. Representative Neurolucida tracing of medium spiny neurons from a saline-treated animal (**A**) and an ethanol-treated animal (**B**), demonstrating the difference in observed branch patterns at 24 h. The scale bar in the bottom right corner of the image represents 10 μm.

**Figure 2:**
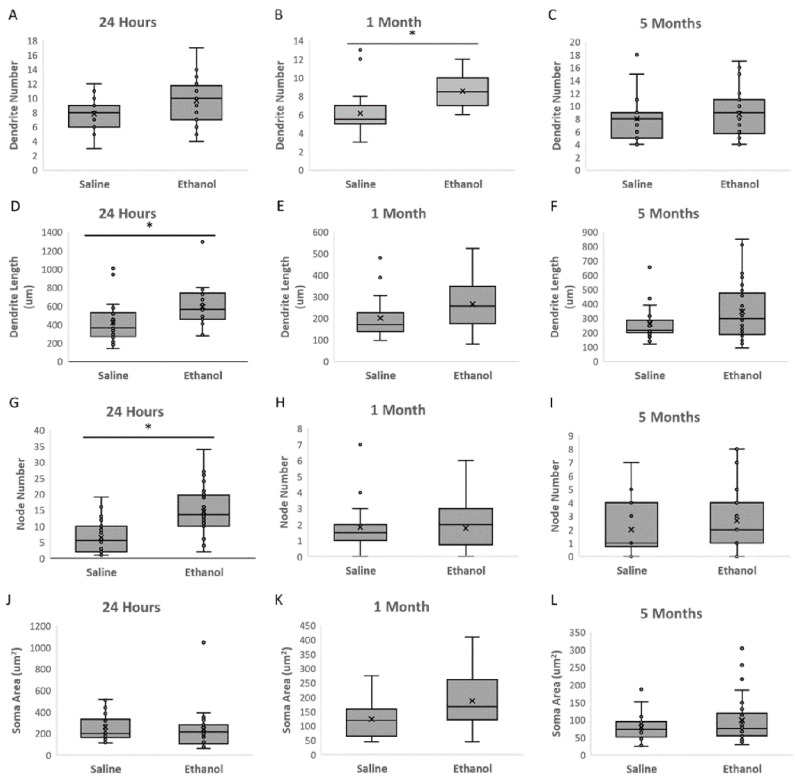
Ethanol treatment during synaptogenesis significantly increases MSN dendritic length and number of nodes after 24 h. (**A**–**C**). Dendritic number was significantly greater in ethanol-treated animals 1 month after exposure (**B**); *p* = 0.002), and it trended towards significance at 24 h (**A**); *p* = 0.065). There was not enough statistical power to conclude that ethanol did not affect dendrite number at 5 months of age (**C**); Power: 0.717). (**D**–**F**). Dendritic length was significantly greater in ethanol-treated animals 24 h after exposure ((**D**); *p* = 0.027), there was not enough statistical power to determine the effects at 1 month (**E**); Power: 0.3805), and there was no significant difference at 5 months of age (**F**); *p* = 0.384). (**G**–**I**). Dendritic node number was significantly greater in ethanol-treated animals 24 h after exposure (**G**); *p* = 0.018), but the number of nodes was not significantly different at 1 month (**H**); *p* = 0.868). There was not enough statistical power to conclude there was no significant node difference at 5 months of age (**I**); Power: 0.6472). (**J**–**L**). The small sample and effect size provides insufficient statistical power to determine if there is an effect of ethanol on soma size. For all experiments, between 6 and 8 medium spiny neurons in the caudate-putamen region of the striatum were traced in 3–5 animals at each treatment level (*n* = 4 saline and *n* = 4 ethanol at 24 h; *n* = 3 saline and 3 ethanol at 1 month; *n* = 3 saline and *n* = 5 ethanol at 5 months). For all panels, error bars represent SEM values. *: significant at *p* < 0.05.

To investigate ethanol-induced alterations in MSNs, neuronal morphology was measured 24 h, 1 month, and 5 months after acute ethanol exposure during synaptogenesis. Representative Neurolucida tracings of MSNs from control and ethanol-treated mice are shown in Figure 1A,B, respectively. As depicted in Figure 2, acute high-dose ethanol administration during the early postnatal period in mice significantly increased MSN dendritic length after 24 h (*p* = 0.027), but this difference appears to be normalized by adulthood (5 months; *p* = 0.384). Furthermore, ethanol-treated MSNs had significantly more branching nodes after 24 h (*p* = 0.018), but by the 1-month timepoint, there was no longer a significant difference in nodes (*p* = 0.868). For soma size, the small sample and effect size provides insufficient statistical power to determine if there is an effect of ethanol at any timepoint examined (Figure 2J–L). Despite the observed increases in dendritic complexity, ethanol treatment does not significantly increase the actual number of MSNs dendrites at 24 h (though it trends toward significance at *p* = 0.065). At 1 month of age, ethanol does significantly increase the number of dendrites (*p* = 0.002). Though the number of dendrites appeared to normalize at 5 months, the limited sample size for this study provides insufficient statistical power to detect ethanol effects on the number of dendrites at this time point.

### 2.2. Dendritic Spine Analysis following Ethanol Administration

**Figure 3:**
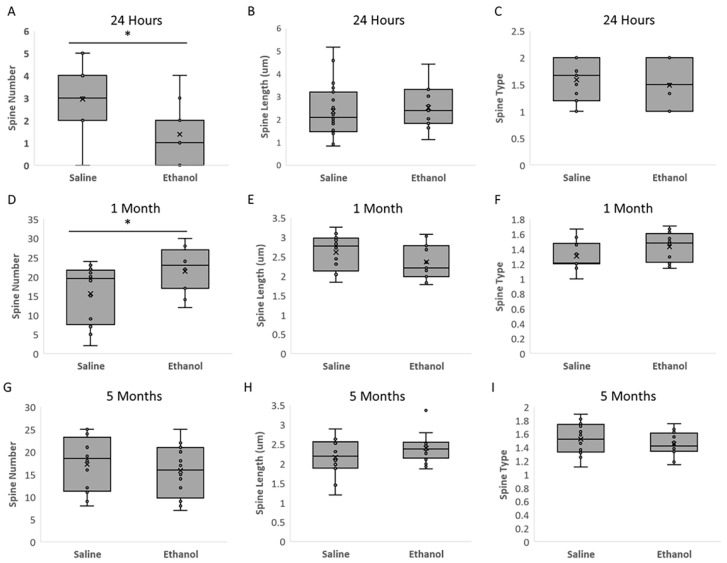
Acute high-dose ethanol administration during synaptogenesis significantly affects dendritic spine number. (**A**–**C**). Twenty-four hours post-ethanol administration: (**A**). number of dendritic spines was significantly decreased in ethanol-treated animals 24 h after exposure (*p* = 0.002), but there was not sufficient statistical power to determine if there was an effect on spine length (**B**); Power: 0.744) or spine type (**C**); power: 0.7003). (**D**–**F**). One-month post-ethanol administration: (**D**). number of dendritic spines was significantly increased in ethanol-treated animals 1 month after exposure (*p* = 0.030), but there was no significant effect of treatment on spine length (**E**); *p* = 0.944). There was not sufficient statistical power to determine if there was an effect on spine type (**F**); Power: 0.4577). (**G**–**I**). Five months post-ethanol administration: No significant differences were detected between ethanol and saline groups 5 months after treatment for spine number (**G**), spine length (**H**), or spine type (**I**), but there was insufficient statistical power to determine the impact of ethanol treatment (power: 0.4496, 0.4577, and 0.5183, respectively). For spine analysis, 3–5 animals were used in each treatment group (*n* = 5 saline and 4 ethanol at 24 h; *n* = 3 saline and *n* = 3 ethanol at 1 month; *n* = 3 saline and *n* = 4 ethanol at 5 months). *: significant at *p* < 0.05.

To determine whether ethanol treatment affects dendritic spine number, length, or type, confocal microscopy was used to analyze Golgi-stained brain sections from ethanol and saline-treated mice. As shown in Figure 3, ethanol treatment significantly decreased the MSN dendritic spine number at 24 h, but increased the number of spines by 1 month of age. Though these spine differences appear to resolve by 5 months of age, there is insufficient statistical power to deduce ethanol’s impact in adulthood (Figure 3A,D,G). We did not detect a significant difference in spine length, but there is insufficient statistical power to determine the relationship between treatment and spine length in all but the 1-month post exposure group (*p* = 0.944), which showed no difference (Figure 3B,E,H). We also did not detect a significant difference in spine type between ethanol and saline treated groups, but there is insufficient statistical power to determine the impact of ethanol treatment on spine type (Figure 3C,F,I).

### 2.3. Protein Analysis

**Figure 4:**
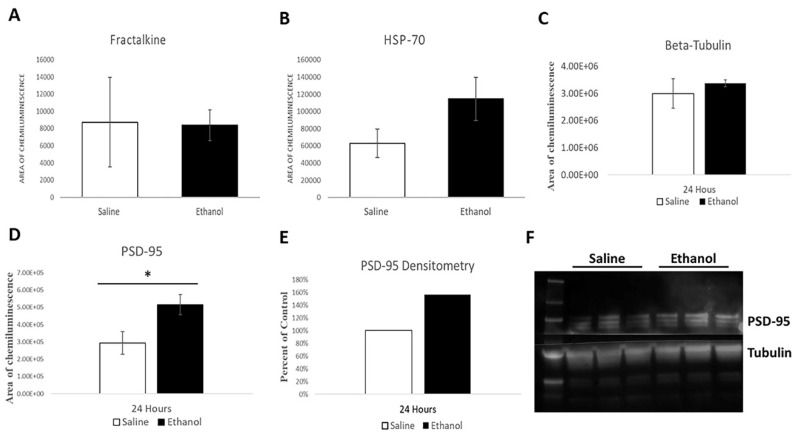
Protein analysis in the striatum of mice 24 h following saline or ethanol administration. (**A**). Fractalkine levels are not affected by ethanol treatment (*p* = 0.477). (**B**). Hsp-70 is increased in response to ethanol treatment, but this difference is not significant (*p* = 0.084). (**C**). Beta-tubulin levels are not affected by ethanol treatment (*p* = 0.281). (**D**). PSD-95 is increased in response to ethanol-treatment (*p* = 0.033). (**E**). Western blot analysis of PSD-95 protein in mouse striatum, normalized with beta-tubulin (*n* = 6 mice for both ethanol and saline samples). (**F**). Representative Western blot of PSD-95 and tubulin proteins from mouse striatal tissue. Each lane represents a striatal sample from a different mouse. For all panels, error bars represent SEM values. *: *p* < 0.05.

To investigate the mechanism by which ethanol is causing such an acute change in MSN dendritic morphology, simple Western blots were used to assess protein levels in striatal samples of saline and ethanol-treated animals 24 h after ethanol administration. Because microglia play an important role in synaptic pruning, levels of the microglia recruitment marker, fractalkine, were analyzed. Fractalkine is expressed by neurons and received by microglia and is known to play a role in the developmental pruning of neurons. Therefore, significant changes in fractalkine level could be responsible for the ethanol-induced changes in spine number. However, as shown in Figure 4A, no differences in fractalkine level were observed following ethanol treatment (*p* = 0.477).

As an alternative mechanism for the ethanol-induced dendritic branching, the levels of Hsp70 were examined. Hsp70 is known to facilitate the formation of cytoskeletal components, protect them during stress, and impact neurite outgrowth. As shown in Figure 4B, although ethanol did not produce a statistically significant difference in Hsp70 levels, there is a marginal trend towards being elevated 24 h after ethanol treatment (*p* = 0.084).

**Figure 5:**
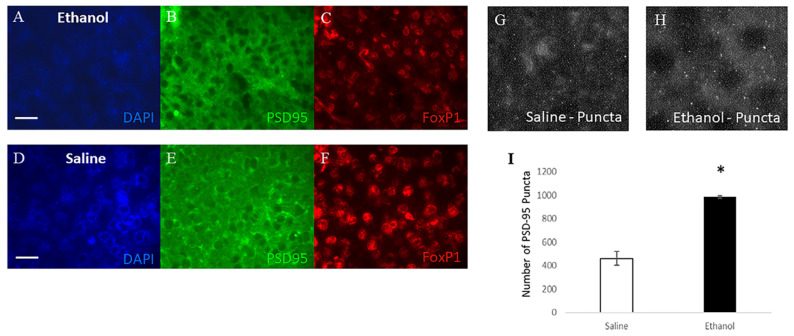
Ethanol treatment increases the level of PSD-95 found in the striatum 24 h after exposure. (**A**–**F**). Immunofluorescence images taken at 400× magnification; images left to right of DAPI, PSD-95, and FoxP1 staining. DAPI labels nuclei, and FoxP1 is a marker for MSNs. (**A**–**C**) depict ethanol-treated animals, while D–F depict saline-treated animals (Scale bar = 50 µm). (**G**–**H**) depict examples of PSD-95 immunofluorescence images used for counting puncta. (**I**). Quantification of PSD-95 puncta in immunofluorescence images (461.46 ± 58.38 saline, 984.17 ± 10.0 ethanol, *: *p* = 0.010).

Because spine numbers are reduced 24 h after ethanol administration, and glutamatergic synapses are found on dendritic spines, the localization of postsynaptic density-95 (PSD-95) was assessed as a marker of active glutamatergic inputs. PSD-95 accumulates at synapses, thereby forming distinct puncta that are visible using immunofluorescence. As shown in Figure 4D–F, total levels of PSD-95 protein are increased 24 h following ethanol treatment in the caudate/putamen, both by simple and standard Western blot technique. Beta-tubulin levels were not affected by ethanol treatment (Figure 4C), and were therefore, used as a loading control to normalize the signals of other proteins. Ethanol treatment also increased the number of PSD-95 puncta in the striatum 24 h after administration (461.46 ± 58.38 saline, 984.17 ± 10.0 ethanol, *p* = 0.010, Figure 5). For immunofluorescence experiments, FoxP1 was used as a marker to identify MSNs and verify that puncta were counted in the correct cell population. This indicates that the morphological differences identified at the 24 h time point correlate with increased levels of the synaptic marker, PSD-95, in the striatum.

## 3. Discussion

The present study investigated the effects of acute ethanol exposure in neonatal mice (P6) on the morphology of dorsal striatal medium spiny neurons (MSNs) immediately, in adolescence, and in adulthood. Our results show that a single ethanol intoxication event induces structural and molecular changes in MSNs compared to saline-treated animals just 24 h later (at P7). Changes include an immediate increase in both MSN dendritic length and dendritic arborization in the ethanol treated animals, but a rapid decrease in spine number relative to the saline control group.

In adolescence (1 month of age), both the number of dendrites and the number of spines were higher in the animals treated with ethanol; however, these differences normalized to control levels by adulthood (5 months of age). We view the immediate dendrogenesis (an increase in dendrite length and node number) following ethanol administration as the cell mobilizing in response to ethanol injury, and we interpret the higher number of dendrites and spines in adolescence as a compensatory response, followed by apparent normalization in adulthood, although statistical power limitations prevent the conclusion that ethanol-induced differences are actually resolved by adulthood.

It is known that ethanol administration can alter MSN morphology; however, the literature reports conflicting findings regarding ethanol branching, particularly in vitro, where ethanol has been shown to inhibit neurite outgrowth by inhibiting PKC and ERK1/2 activation in prenatal rat hippocampal neurons [10], while a separate experiment showed enhanced neurite outgrowth in cerebellar neurons at similar ethanol concentrations. Therefore, in vivo ethanol studies assessing dendrogenesis are an important contribution to this field.

Previous work by Susick et al. found that striatal neurons exposed to an acute ethanol intoxication event (P5-7) had significantly less developed dendrites than the control group at one month of age [6], whereas we found more dendritic branches at one month of age following ethanol exposure. Although the timing and route of ethanol exposure is consistent between these studies, there are differences in experimental design that may partly account for this discrepancy in results. Firstly, while Susick et al. administered a single dose of ethanol (2.5 g/kg) to P5-7 pups, we administered two doses of ethanol (2.5 g/kg) spaced two hours apart. When a 2.5 g/kg dose is repeated subcutaneously after 2 h, as in our paradigm, C57BL/6 pups achieve a mean blood ethanol content (BEC) 1 h after the second injection of 472 (±16) mg/dL [3]. This is considerably higher than the BEC of ~250 mg/dL that was achieved by Susick et al. using a single ethanol administration [6]. Furthermore, whereas Susick et al. used only male mice in their study, our study pooled both males and females. Gender differences have been identified in the metabolism of ethanol and the effect of ethanol administration on behavior in C57/BL6 mice [11]. Lastly, Susick et al. only examined tissue 30 days after treatment, whereas we examined tissues after 24 h, 1 month, and 4 months, with the most significant findings at 24 h post-ethanol administration, a timepoint they did not investigate.

Our demonstration of clear immediate changes in MSN morphology is useful in the exploration of ethanol’s impact within a living system, as it is likely that complex striatal interconnections mediate the neuronal reaction to ethanol differently in vivo compared to when neurons are isolated away from their networks. Our results also provide additional directional evidence to this phenotype, showing that ethanol enhances branching in the time period right after exposure. When viewed in the light of other published studies, our results show that the immediate striatal response to ethanol exposure during the postnatal period may be different from the long-term response to ethanol.

### 3.1. Dendrogenesis and HSP-70

MSNs are remarkably plastic, even in adult animals, especially in the morphology of their dendrites [12]. However, the mechanism for how ethanol causes such an acute change in MSN dendritic morphology is not known. One explanation for our arborization findings could be that ethanol induces the branching within the dendrites. Neurite outgrowth and spine morphogenesis are mediated by reorganization of actin components and the microtubule cytoskeleton. As such, we examined levels of Hsp70, which is a heat shock protein (70 kDa) able to facilitate the formation of cytoskeletal components and protect them during stress. In the postnatal period, heat shock proteins continue to protect the developing CNS from unfavorable conditions and are able to impact neurite outgrowth [13].

Although we found no statistically significant difference in HSP70 levels, there is a marginal trend towards being elevated 24 h after ethanol treatment. Normally, HSP70 is present at the synapse, associated with the post synaptic density, and can colocalize with PSD95 [14], but Hsp70 also localizes to the synapse upon stress [15]. Acute administration of 5 g/kg ethanol results in an increased level of Hsp70 in various brain regions in rats, including the striatum [16]. Although the specific function of the PSD-associated HSP-70 is not known, it could possibly facilitate local synthesis of the new proteins required for neural remodeling and neurite outgrowth or stabilize synapses during periods of structural plasticity, including that seen immediately after ethanol administration. Changes here likely involve actin/microtubule regulator molecules, although the mechanisms by which immediate changes occur after ethanol administration are still under investigation, and further research is warranted on heat shock family proteins.

### 3.2. Fractalkine Signaling and Neuronal Pruning

Another possible explanation for the observed branching differences is that ethanol could inhibit a natural pruning process that takes place during neurodevelopment. To investigate a potential change in the pruning of neurons, we examined a marker of microglia recruitment. The microglia play a key role in a number of important processes in the developing brain, including dendritic spine pruning and the clearing of apoptotic cells during development [17,18]. However, it has been established in the literature that ethanol treatment activates microglia [19,20], so instead, we assessed levels of fractalkine, a molecule expressed by neurons and received by microglia, which plays a normal role in the developmental pruning of neurons and can recruit microglia to a particular location. Not only is fractalkine signaling a mediator of microglia–neuron communications during synaptic plasticity, but fractalkine is also released after neuronal stress and has a role in the response to ethanol injury [21].

During the postnatal period of development, fractalkine is responsible for circuitry refinement via synaptic engulfment and timing of microglia recruitment, via glutamatergic synapse formation and delayed microglia-synapse interaction during development [22,23,24]. Although ethanol can suppress the expression of the fractalkine ligand CX3CL1 in microglia in a neonatal mouse model of FASD [25], our striatal protein quantification found no significant differences in fractalkine levels. Our results align with a similar previous study that found no changes in fractalkine mRNA expression in a similar postnatal ethanol paradigm [21].

### 3.3. PSD-95 as a Neuroprotector

We next investigated the role of PSD-95 as both a neuroprotective molecule and as a potential marker for active new synapses on these dendritic branches. Proteins such as PSD-95 that are primarily involved in synaptic functions can also play developmental roles in shaping branch patterns. PSD-95 is a postsynaptic scaffolding protein that acts as an organizer for signaling components at the synapse, but also promotes dendritic spine maturation [26,27] and regulates synaptic transmission [28].

Our results show that ethanol increases PSD-95 levels in the striatum after 24 h. This is consistent with previous research, which found that spine reduction may actually result in an enhancement of the amount of PSD-95 present on the dendritic shaft [29]. Our results are also in line with a similar study that found an immediate early rise in PSD-95 levels after a single acute ethanol exposure [30].

As a synaptic protein, PSD-95 can balance the interplay between the dopamine and glutamate systems in synapses by regulating the localization, trafficking, and stabilization of both glutamatergic NMDA receptors and dopamine receptors [31]. Dopamine alters the sensitivity of other neurons to neurotransmitters, particularly glutamate [32]. Overactivity of dopamine D1 receptors and glutamatergic NMDA receptors can lead to NMDAR-mediated excitotoxicity, causing cell death and neurological phenotypes. PSD-95 can act as a neuroprotector in the striatum that can limit D1 activity and inhibit the crosstalk between D1 and NMDA receptors to limit the effects of excitotoxicity.

PSD-95 knock-out mice show striatal NMDA receptor hyperactivity and MSN cell death [33], as well as increased sensitivity to the sedative/hypnotic effects of ethanol [34]. Therefore, it is possible that the increased PSD-95 seen in our model is an immediate neuronal compensatory response to the ethanol, possibility elevated to protect against excitotoxicity effects in the striatum.

### 3.4. PSD-95 in Development

In its developmental role, PSD-95 can shape the dendritic arbor and spines via microtubule-dependent mechanisms [35] in a manner specific to immature neurons [36]. Research suggests that as the neuron matures, increasing levels of PSD-95 provide an environment that is more friendly to the development of new spines than new branches, and dramatic neuronal morphology changes become more infrequent [37]. In addition, overexpression of PSD-95 increases spine maturation [26] which collectively supports a role for PSD-95 during multiple phases of neuronal development, all of which could be affected by ethanol-induced expression changes, depending on the exposure window [38].

In light of this, it is likely that PSD-95 plays a role in the changes in spines that we see, but is unlikely to be the mechanism causing the increased immediate neuronal branching identified after 24 h. In fact, high PSD95 expression levels decrease branching, so the mechanism underlying the immediate branching phenotype must be robust enough to counter the PSD95 role in decreasing neurite outgrowth. An important next research step will be to isolate ethanol’s effect on the developmental role of PSD-95 from PSD-95′s more immediate role in synaptic function. In addition, an examination of later timepoints could provide information about how long the PSD-95 elevation persists.

### 3.5. Adolescence and Ethanol Withdrawal Effects

At 1 month of age (3 weeks of withdrawal), we observed an increase in MSN dendrite number and spine number in ethanol treated animals. Previous research has demonstrated a pattern of increased dendritic modifications after ethanol withdrawal using models of chronic ethanol exposure. Researchers found that after 5 months of chronic ethanol administration and 2 months of withdrawal, dendrite segments increased in hippocampal CA1 neurons as the cells compensated for the ethanol-induced developmental retardation [39]. A separate study found an immediate decrease in spines in the nucleus accumbens following one day of alcohol withdrawal after chronic administration of ethanol in rats, followed by increased dendritic length in the context of unaltered spine density in nucleus accumbens core MSNs at 7 days of withdrawal. Spine loss may represent ethanol-dependent pruning of spines, where most active synapses are retained following ethanol exposure [40].

The processes occurring during ethanol intoxication are different from those during the withdrawal period. Even though acute doses of ethanol inhibit NMDA receptors in adults, neurons can adapt by increasing the sensitivity or number of NMDA receptors. Once the ethanol is metabolized, this receptor increase can result in excitotoxicity. Early postnatal ethanol exposure in rodents has been shown to increase markers for glutamatergic activity (NMDA1 receptor) in the prefrontal cortex, dorsal striatum, somatosensory cortex, and motor cortex during acute withdrawal (22 or 26 h post ethanol administration) [41]. During normal development, there is a sharp increase in vulnerability to NMDA receptor-mediated excitotoxicity in the third trimester equivalent (P6–P7 in rats), and the associated behavioral deficits can be ameliorated with the administration of an NMDA antagonist 21–33 h after ethanol administration. This explains how ethanol-induced cell death can be excitotoxic, even though ethanol itself inhibits the excitatory activity of the NMDA receptor.

### 3.6. The Importance of Arborization within the Striatum

Our results offer a new perspective on the neural response to ethanol, as we show that neurons exhibit an immediate response to the ethanol intoxication where spines decrease, but dendrites are more arborized. These findings open multiple avenues for further research to determine exactly how and why the neurons respond in this way. We know that the brain is most sensitive to injury during periods of intensive growth [42], and so it is also important to consider the effect that these structural changes will have on cell and circuit functionality.

Although MSN morphology appears to be largely corrected in our paradigm by adulthood, even a transient window of dysregulation in neuroarchitecture could have enormous impacts on the animal, particularly in the striatum, at this developmental timepoint. The striatum is an important signaling hub of the brain, collecting information and distributing it to a number of targets. It receives extensive glutamatergic inputs from the cortex, thalamus, hippocampus, and amygdala. It integrates this information and sends it to the globus pallidus, subthalamic nucleus, substantia nigra, and ventral tegmental area (VTA) (reviewed in [43,44]). The majority of alcohol research within the striatum focuses on the ventral striatum because of its key role in addiction, but we chose to examine the caudate and putamen within the dorsal striatum. While the ventral striatum is involved with cued outcome-mediated behaviors, the dorsal striatum makes contributions to goal-directed and habitual reward-seeking behavior [45].. Acute ethanol can change GABAergic transmission in this area [46], with a net effect of increasing output from the sensorimotor striatum, possibility initiating habit formation and having additional implications for addictive behaviors [8].

Before the dendritic arbor stabilizes in the mature animal, large-scale morphological changes occur in neurons during the first weeks of postnatal development, characterized first by growth of dendritic branches and followed by the pruning of excessive or mistargeted branches [36]. This branched morphology of the neuronal dendritic arbor allows for the induction of synaptic plasticity and enables information to enter, be integrated, and be sent to the rest of the neural circuit. Proper regulation of these processes is crucial for neuronal function and synaptic transmission, and as such, abnormal development of the dendritic arbor contributes to a host of brain disorders [47]. We also know that spines can be differentially regulated by neuronal activity; specifically, dendritic shaft filopodia elongate in response to focal glutamate application [48], which is particularly relevant for the striatum. Alterations in the number and shape of spines contribute to aberrant synaptic and neural signaling, since the distribution pattern of synapses and the properties of each synapse will determine the connections in and out of each MSN [38,49].

In many neurons, the location of a synaptic conductance within the dendritic tree has the ability to profoundly influence the timing and amplitude of the somatic EPSP [50]. Therefore, even in the absence of gross morphology, deficits may be present in high-level sensory and motor processing, as well as complex cognitive thought and behavior.

### 3.7. The Importance of the MSN Early Postnatal Period

Our chosen developmental timepoint is particularly important for immature MSNs, as many MSN electrophysiological characteristics change abruptly between mouse postnatal days 6 and 10. Research indicates the presence of a developmental program that switches MSN activity from an immature low threshold activation state to a high threshold state in the adult during resting conditions that coincides with the emergence of locomotion. This “adult” state is also mirrored in the development of the MSN arborization/spines, an increase in glutamatergic synapses, and the solidifying of nigrostriatal dopaminergic inputs [51]. Of note, these patterns are similar to those seen in the developing cortex, so it is likely that other brain areas have comparable mature patterns taking over at timepoints appropriate to support each brain area’s main function.

Just as alcohol impacts the developmental events that occur at the time of administration, learning and memory processes are also limited by the state of the neurons during the time that acquisition is happening. Among other things, the dorsal striatum is important for the consolidation of memory learning that accrues over many trials [52]. Our lab has investigated both social behavior and learning/memory in mice treated with this acute ethanol developmental paradigm. We found no overt social differences, but the animals exhibit memory deficits on the Barnes maze when learning acquisition occurs during adolescence and retention is tested in adulthood [7,53] This acquisition deficit fits well with our finding of PSD-95 abnormalities, as PSD-95 also appears to be important in coupling the NMDA receptor to pathways controlling synaptic plasticity and learning [28]..

The dendrogenesis and spinogenesis observed immediately after an intoxication event indicate an immediate response to ethanol, but morphological changes also persist into adolescence. Therefore, brief acute exposure to a high ethanol dose during the third trimester equivalent of human pregnancy may have a permanent negative impact on not just the morphology of neurons, but also the behavior and neural functioning of the offspring in adulthood.

## 4. Materials and Methods

### 4.1. Animal Care

Protocols for animal use were approved by the Institutional Animal Care and Use Committee at Hampden-Sydney College. All mice were offspring from timed pregnant C57BL6/J females (Jackson Laboratories), received on the same gestational day and subsequently housed in a temperature-controlled room under 12-h light/dark cycles (lights off at 6 p.m.), with ad libitum access to food and water. A total of 6 C57/Bl6 timed pregnant females were ordered from Jackson labs. Each pup within a litter was randomly assigned to either the saline or ethanol treatment. Cumulatively for all experiments, *n* = 19 saline pups and *n* = 20 ethanol pups were assessed.

### 4.2. Ethanol Treatment Paradigm

C57BL6/J mice were exposed to a high acute ethanol dose on postnatal day 6 (P6) by dorsal subcutaneous injection of a 20% ethanol solution (2.5 g/kg dose) or saline control, followed by a second injection two hours later [7]. Pups were individually randomly assigned a treatment condition and were returned to their mothers between and after injections.

When a 2.5 g/kg dose is repeated subcutaneously after 2 h, as in our paradigm, C57BL/6 pups achieve a mean blood ethanol content (BEC) 1 h after the second injection of 472 (±6) mg/dL, with an alcohol clearance rate of 283 mg/dL/h. This blood ethanol level is within the range that a human fetus might be exposed to after maternal ingestion of a moderate to heavy dose of ethanol [3].

Mice were anesthetized deeply with isoflurane, followed by cervical dislocation, at either 24 h, 1 month, or 5 months following exposure. Brains were harvested and halved at the longitudinal fissure. One entire hemisphere was flash frozen using isopentane and frozen at −80 °C until use, and the striatum was dissected out from the other hemisphere before freezing. The 24-h time point animals came from 4 litters of timed-pregnant females injected at P6 and euthanized at P7. The one- and five-month time point animals came from 4 separate litters injected at P6 and euthanized at P30 and 5 months, respectively.

### 4.3. Golgi Staining and Neuronal Morphology

Twenty-four-hour brains were coronally sectioned using a sliding microtome at 60 µm and Golgi processed as described in Brunjes and Kenerson [54]. One-month and 5-month brains were sectioned using a cryostat at 60 µm and Golgi processed using the FD GolgiStain kit (FD Neurotechnologies, Inc., Columbia, MD, USA).

Camera Neurolucida (2015) tracings were prepared using the 40× lens. For analysis, all selected neurons had to meet rigid criteria: the neuron had to be well-impregnated, the branches could not be obscured by other neurons, or other precipitates, there could be no visibly broken processes on the neuron, it had to be located within the caudate/putamen region of the striatum (between Bregma +1.54 and −1.94 mm), and it had to visually resemble a typical medium spiny neuron.

For neuronal morphology, between 6 and 8 medium spiny neurons in the caudate-putamen region of the striatum were traced in 3–5 animals at each treatment level (*n* = 4 saline and *n* = 4 ethanol at 24 h; *n* = 3 saline and 3 ethanol at 1 month; *n* = 3 saline and *n* = 5 ethanol at 5 months).

Following the tracing, the NeuroExplorer program was used to quantify the morphological data. Four parameters were assessed, including the number of dendrites, number of nodes, total length of dendrites per neuron, and cell body size. The number of dendrites was defined as the number of projections that were extending directly from the soma. The number of nodes was the count of the branch points on those dendrites. Dendritic length was the total length of all projections outside the soma.

### 4.4. Dendritic Spine Analysis

Image stacks were taken on a Zeiss confocal scope using the 100× objective lens. For each animal, a section of apical dendrite was measured on first order branches (located immediately between the soma and the first dendrite branch point) on 4 different neurons. The average number of spines were counted (*n* = 5 saline and 4 ethanol at 24 h; *n* = 3 saline and *n* = 3 ethanol at 1 month; *n* = 3 saline and *n* = 4 ethanol at 5 months). All visible spine heads protruding from the apical dendrite were counted beginning at 40 µms from the soma and ending at 80 µms from the soma to obtain a spine density. The length of individual spines was measured from the distal end of the spine to the point where the spine attached to the dendrite and an average spine length was calculated for each neuron. Spines were simply classified as (1) filipodia >2 µm or (2) non-filopodial spines <2 µm in length [55].

### 4.5. Immunostaining

Following euthanasia, mouse brains were immediately immersed in 4% paraformaldehyde for a week, embedded in paraffin, and sectioned at 5 µm. Sections were dewaxed and rehydrated by incubating 5 min Clear-Rite 3, 5 min 100% EtOH, 2 min 95% EtOH, 2 min 70% EtOH, and 2 min distilled water. Antigens were retrieved by boiling 20 min in citrate buffer (10mM citric acid, 0.05% Tween 20, distilled water). Slides were then cooled in an ice bath and placed in 1x PBS 3 × 5 min at room temperature (RT). Sections were blocked with 10% HING, 0.1% Triton in 1x PBS for 10 min at RT. Slides were then washed in 3 changes of 1x PBS + 0.05% Tween 20 for 10 min each at RT. Primary antibodies were diluted in 1x PBS (rabbit anti-PSD-95 Abcam ab18258 and mouse anti-FoxP1 Abcam JC12; 1:500) and incubated overnight at 4 °C. Sections were washed using the same washing solution as before at RT. Fluorescent secondary antibodies (AB150116 AlexaFluor 594; AB150077 AlexaFluor 488; Abcam) were diluted 1:200 in 1x PBS and allowed to incubate for 2 h at RT. Sections were washed as before for 3 × 10 min. Coverslips were mounted in mounting media containing DAPI, sealed, and allowed to dry. To confirm the presence of PSD-95 within MSNs, FoxP1 was used as an MSN marker and used to locate the striatum within each brain. DAPI was used as a control to confirm the presence of cell bodies colocalized with FoxP1.

### 4.6. Fluorescent Imaging and Quantification

Imaging was performed using a Zeiss Axioskop 40 microscope, and images were taken with an AxioCam MRc camera using the program AxioVision LE 4.8. Ten field of view images were taken at 40× magnification, puncta were quantified manually and averaged for each animal. The results were then averaged for each treatment. For quantification, fluorescent image files were coded alphabetically to conceal treatment group and given to a separate researcher to complete the PSD-95 puncta counting. Areas of punctate staining were counted if they were a small bright dot, generally excluding the cell body, and colocalized with FoxP1.

### 4.7. Protein Analysis

For protein analysis, brains were removed from storage in −80 °C and the striatum was lysed in neuronal protein extraction reagent (N-PER; ThermoFisher Scientific, Waltham, MA, USA). Simple Westerns (RayBioTech, Peachtree Corners, GA, USA) were performed for PSD-95 (antibody ab18258, Abcam), fractalkine (BAF472, R&D Systems, Minneapolis, MN, USA), HSP70 (AF1663, R&D Systems) and tubulin (NB600-936, Novus Biologicals, Littelton, CO, USA). For traditional Western blots, protein samples were run on 4–20% polyacrylamide gels. Proteins were transferred overnight to PVDF membrane using wet transfer procedures. Blocking and all washes were performed in TBS containing 5% dry milk and 0.5% Tween20. Polyclonal anti-PSD-95 antibody (#2507, Cell Signaling Technology, Danvers, MA, USA) was used at a 1:1000 dilution to detect PSD-95 protein. Antigen-antibody complexes were further reacted with HRP-conjugated secondary antibody (Jackson Immuno-Research Laboratories, West Grove, PA, USA) and detected by chemiluminescence (SuperSignal Pico reagent, Pierce, Rockford, IL, USA). The blots were stripped and re-reacted with monoclonal anti-tubulin antibody (1:1000 dilution, #T7451 Sigma-Aldrich, St. Louis, MO, USA, to normalize the signals. Chemiluminescent images were captured using a ChemiDoc Imager (Bio-Rad, Hercules, CA, USA and signals were quantified using ImageLab software (Bio-Rad). For each animal analyzed, the chemiluminescent signal for PSD-95 was normalized to its respective Tubulin chemiluminescent signal.

### 4.8. Statistics

Analyses sought to examine the impact of treatment on multiple dependent variables at each separate time point. Data were analyzed using generalized linear mixed models (GLMMs) in R [44] using the ‘glmmTMB’ package [45], with ethanol treatment as a fixed-factor and maternal family as a random factor. When possible, data were transformed to meet parametric assumptions. When this was not possible, data distribution was determined using AIC comparisons and the GLMM used the distribution of best fit. Boxplots indicate the median and quartile values of treatment groups.

Post hoc power analyses were conducted for all timepoints using the statistical software G*Power version 3.1.9.7 [46]. Power below 0.80 was considered insufficient for results interpretation. 

For fluorescence, protein expression levels were analyzed by averaging the number of areas of punctate staining (*n* = 3 animals in each treatment level, using an average value from 10 images taken from each animal) for immunostaining and statistical significance was determined using unpaired *t*-tests. Western analysis used densitometry normalized to b-tubulin expression levels (*n* = 3), and statistical significance was determined using unpaired *t*-tests.

## Data Availability

Data generated during the study are contained within the article.
